# Distribution of *fragile X mental retardation 1* CGG repeat and flanking haplotypes in a large Chinese population

**DOI:** 10.1002/mgg3.128

**Published:** 2014-12-15

**Authors:** Wen Huang, Qiuping Xia, Shiyu Luo, Hua He, Ting Zhu, Qian Du, Ranhui Duan

**Affiliations:** The State Key Laboratory of Medical Genetics & School of Life Sciences, Central South UniversityChangsha, 410078, Hunan, China

**Keywords:** *FMR1*, CGG repeat pattern, haplotype, Chinese population

## Abstract

Fragile X syndrome is mainly caused by a CGG repeat expansion within the 5′ UTR of the *fragile X mental retardation 1* (*FMR1*) gene. Previous analyses of the *FMR1* CGG repeat patterns and flanking haplotypes in Caucasians and African Americans have identified several factors that may influence repeat instability. However, the CGG repeat patterns and distribution for FRAXAC2 have not yet been investigated in mainland Chinese. We surveyed the CGG repeat lengths in 1113 Han Chinese (534 males and 579 females), and the CGG repeat patterns of 534 males were determined by sequence analysis. We also explored the flanking haplotypes (DXS548-FRAXAC1-FRAXAC2) in 566 unaffected and 28 unrelated fragile X Chinese males. The most frequent alleles for DXS548 and FRAXAC1 were identical between our Chinese population and other Asian populations. We identified several low-abundance alleles for DXS548 and FRAXAC1 not found in previous studies in mainland Chinese and Taiwanese cohorts. The most frequent allele was (CGG)_29_ followed by (CGG)_30_, and the most frequent patterns were 9 + 9 + 9, 10 + 9 + 9, and 9 + 9 + 6 + 9, similar to those in Singaporeans. We identified only one premutation female carrier with 89 CGG repeats in the 1113 Han Chinese. A few associations between the CGG repeat patterns and flanking haplotypes were determined in this study. In general, the Chinese population had a smaller number of alleles and lower expected heterozygosity for all three STR markers and FRAXA locus when compared with Caucasians and African Americans. We identified a novel haplotype 7-3-5 + that is significantly associated with the full mutation.

## Introduction

Fragile X syndrome (FXS) is the most common form of inherited intellectual disability and the most common monogenic cause of autism, with an estimated incidence of 1 in 7000 males (Boyle and Kaufmann 2010; Hunter et al. 2014). FXS is predominately due to an expansion of a CGG repeat located in the 5′ UTR of the *fragile X mental retardation 1* (*FMR1*) gene (MIM# 309550) (Kremer et al. 1991; Oberle et al. 1991). Normal alleles contain 6–44 repeats and are usually interspersed in every 9 or 10 repeats with an AGG (Eichler et al. 1994; Hirst et al. 1994; Kunst and Warren 1994; Snow et al. 1994; Zhong et al. 1995). Intermediate alleles with 45–54 repeats carry some risks for expansion to premutations on transmission (Nolin et al. 2011). Premutation alleles have 55–200 repeats, which are prone to expand into methylated full mutations with more than 200 CGG repeats during germline transmission (Hagerman and Hagerman 2002). Expansion to the full mutation leads to hypermethylation and silencing of *FMR1*, resulting in the absence of its protein product, FMRP, which causes FXS (Wang et al. 2012).

Large-scale population studies in Caucasians and African Americans identified several factors that may be involved in CGG repeat instability, such as the number and position of AGG interruptions, purity of the CGG repeat at the 3′ end, and flanking haplotypes (Eichler et al. 1996; Crawford et al. 2000a,b; Peprah et al. 2010). Analysis of the CGG repeat patterns and flanking haplotypes (DXS548-FRAXAC1-FRAXAC2) in 214 normal and 16 premutation Caucasians demonstrated two distinct mutational pathways predisposing CGG repeat to expansion. One pathway involves a single fragile X haplotype (2-1-3) that maintains two AGG interruptions and slowly progresses toward instability in a stepwise manner. The second pathway involves frequent and recurrent loss of AGG interruptions occurring on fragile X haplotypes 6-4-5 and 6-4-4 (Eichler et al. 1996). Crawford and colleagues identified a separate mutational pathway that the lack of a proximal AGG interruption may increase CGG repeat instability by analyzing the CGG repeat patterns of 213 unaffected African Americans (Crawford et al. 2000b). They also examined the CGG repeat size and flanking haplotypes (DXS548-FRAXAC1-FRAXAC2) in 637 unaffected and 63 fragile X African Americans, along with 721 unaffected and 102 fragile X Caucasians (Crawford et al. 2000a). Haplotype (DXS548-FRAXAC1) analysis has been performed in mainland Chinese (*n* = 206), Taiwanese (*n* = 100), Hong Kongers (*n* = 217), Thais (*n* = 125), and Indonesians (*n* = 1043), together with fragile X patients from mainland China (*n* = 24), Taiwan (*n* = 28), and Thailand (*n* = 25) (Poon et al. 1999; Zhong et al. 1999; Faradz et al. 2000; Limprasert et al. 2001; Tzeng et al. 2005). Studies have been conducted to evaluate the CGG repeat patterns in Japanese (*n* = 21), Asians (*n* = 144), and Taiwanese (*n* = 78) (Chen et al. 1997; Hirst et al. 1997; Chiu et al. 2008). The CGG repeat patterns and flanking haplotypes (DXS548-FRAXAC1-FRAXAC2) have been investigated in 454 normal Singaporeans (Zhou et al. 2006).

Han Chinese are divided into southern Han and northern Han, constituting 92% of the population of mainland China and ∼19% of the entire global human population (Wen et al. 2004; Zhou et al. 2011). The CGG repeat patterns and distribution for FRAXAC2 have not yet been investigated in such a large population. We examined the CGG repeat lengths in 1113 unaffected Han Chinese (534 males and 579 females), and the CGG repeat patterns of the 534 males were determined by direct sequencing. We also analyzed the flanking haplotypes (DXS548-FRAXAC1-FRAXAC2) in the 566 unaffected and 28 unrelated fragile X Chinese males.

## Materials and Methods

### Study samples

This study was approved by the Ethics Committee of the State Key Laboratory of Medical Genetics, Central South University. Blood samples from 1145 unaffected individuals (566 males and 579 females) from southern Han and northern Han were collected with informed consent. Individuals were widespread across all 31 provinces of mainland China (a province is the highest level of Chinese administrative division and territorial unit, analogous to a state in the USA). Twenty-eight unrelated Chinese fragile X males were derived from the State Key Laboratory of Medical Genetics, Central South University, with complete informed consent of the patients and their parents. Genomic DNA was extracted from peripheral blood leukocytes using the proteinase-K-chloroform method.

### STR markers genotyping

DXS548, FRAXAC1, and FRAXAC2 were amplified by polymerase chain reaction (PCR) using AmpliTaq Gold enzyme (Applied Biosystems, Shanghai, China). The primers for DXS548, FRAXAC1, and FRAXAC2 were as described previously (Richards et al. 1991; Verkerk et al. 1991). Thermal cycling was performed in a C1000™ Thermal Cycler with an enzyme activation step at 95°C for 5 min, followed by 32 cycles of 95°C for 45 sec, 62°C for 45 sec, and 72°C for 45 sec, and a final extension at 72°C for 7 min. Analysis of *FMR1* CGG repeat length was conducted as previously described (Saluto et al. 2005). Each PCR product of the three STR markers and *FMR1* CGG repeat was analyzed on the ABI 3100 Genetic Analyzer (Applied Biosystems). In total, 224 females with the presence of a single amplicon peak were further examined by Southern blot, to exclude possible large expanded CGG alleles that cannot be amplified by PCR analysis.

### Sequence analysis of *FMR1* CGG repeat

A *FMR1* CGG repeat was amplified with the primers (5′-GCGCTCAGCTCCGTTTCGGTTTCACTTCC and 5′-CCCAAGTCCAGTCCTTCCCTCCCAACAACA) using LA Taq (TaKaRa), and the PCR products were analyzed by sequencing. Thermal cycling was as follows: denaturation at 96°C for 3 min and 10 cycles of 98°C for 20 sec, 65°C for 45 sec, and 72°C for 3 min, followed by 22 cycles of 98°C for 20 sec, 68°C for 3.5 min, and a final extension at 68°C for 10 min.

### Southern blot analysis

Five micrograms of blood DNA was digested with EcoR I/Eag I, and hybridized with the digoxigenin-labeled probe StB12.3 (11669940910; Roche, Shanghai, China) as described (Gold et al. 2000).

### Statistical analysis

Chi-square test was used to compare distributions for STR markers, flanking haplotypes, and FRAXA locus between populations as previously described (Peprah et al. 2010). The expected heterozygosity (EH) value was calculated with the formula, EH = 1 − Σq^2^ (q denotes the frequency for each individual allele at STR markers or FRAXA locus).

## Results

### STR markers in unaffected Chinese population

**Figure 1:**
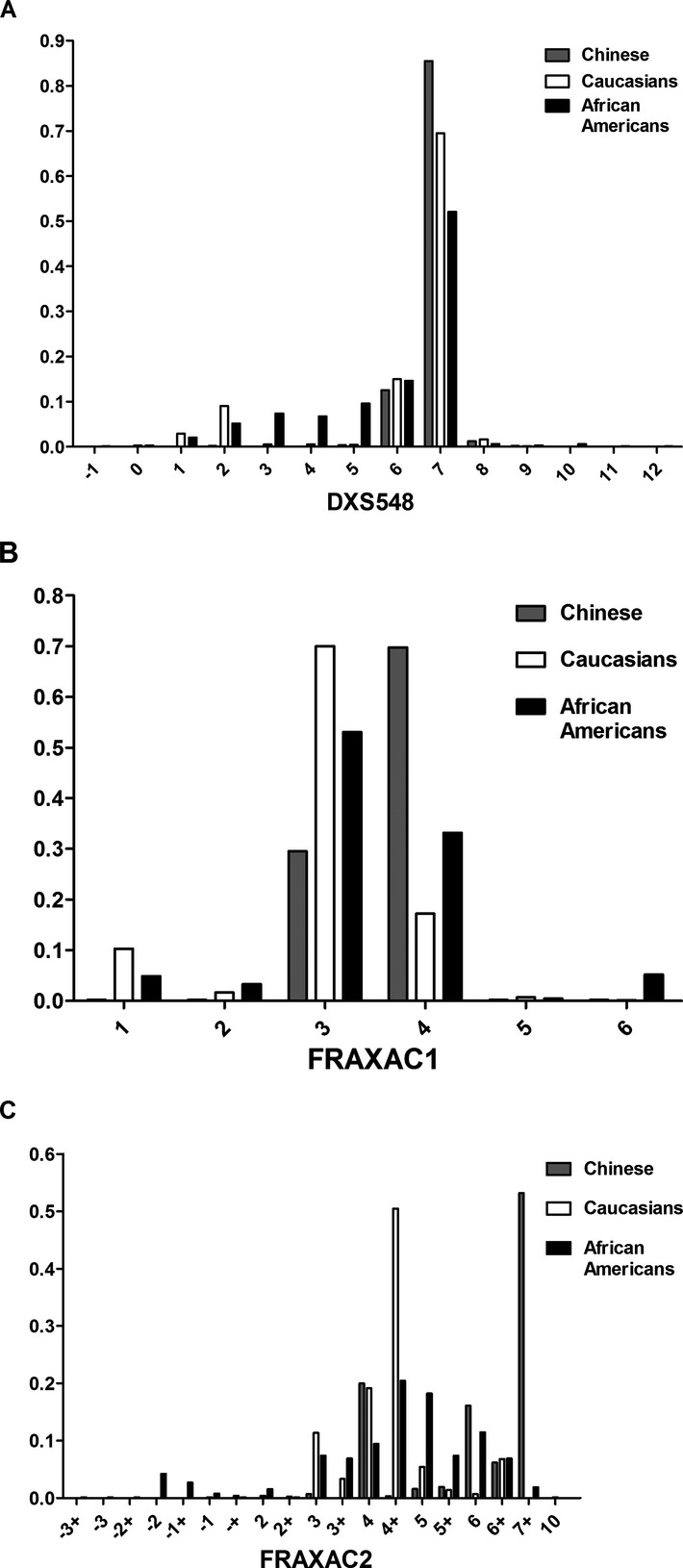
Frequency distribution of STR markers flanking the *FMR1* gene. DXS548 (A), FRAXAC1 (B), and FRAXAC2 (C) allele distributions among the unaffected Chinese (*n* = 566; gray bars), Caucasians (*n* = 721; white bars), and African Americans (*n* = 637; black bars) (Crawford et al. 2000a). *FMR*, *fragile X mental retardation 1*.

We genotyped, 566 unaffected Chinese males for three STR markers (DXS548, FRAXAC1, and FRAXAC2) flanking the *FMR1* gene locus. The nomenclatures of the STR markers are as described previously (Eichler et al. 1996), and all STR-based haplotypes in this study are constructed in the order, DXS548-FRAXAC1-FRAXAC2. We identified six different alleles for both DXS548 and FRAXAC1, and eight different alleles for FRAXAC2 in the Chinese population (Table1). The most frequent alleles in the Chinese population were allele 7 for DXS548 and allele 4 for FRAXAC1. In this study, we discovered allele 2 for DXS548 and allele 1, 2, 5, and 6 for FRAXAC1, which have not been found in previous reports on mainland Chinese and Taiwanese populations (Table1). The most abundant alleles for FRAXAC2 were alleles 7+, 4, and 6 in the Chinese population (Fig.1C). In general, the Chinese population has a smaller number of alleles and a lower EH than Caucasians and African Americans (Table2). With respect to DXS548, the most dominant allele (allele 7) was the same among the three populations (Fig.1A). The most common alleles for FRAXAC1 and FRAXAC2 in the Chinese population differ from that in both Caucasians and African Americans (Fig.1B and C). The allele distributions for all three STR markers were significantly different between the Chinese population and the other two populations (Fig.1A–C).

**Table 1:** Allele distributions for DXS548, FRAXAC1, and FRAXAC2 in unaffected and fragile X Chinese populations

Alleles	Chinese				Taiwanese	
	This study		Zhong et al. (1999)		Tzeng et al. (2005)	
	Unaffected	FXS	Unaffected	FXS	Unaffected	FXS
DXS548						
2	1 (0.002)	1 (0.036)				
4			1 (0.004)			
5	2 (0.004)		3 (0.013)			
6	71 (0.125)	1 (0.036)	30 (0.132)	20 (0.741)	7 (0.070)	
7	484 (0.855)	26 (0.928)	183 (0.806)	6 (0.222)	90 (0.900)	27 (0.964)
8	7 (0.012)		10 (0.044)	1 (0.037)	2 (0.020)	1 (0.036)
9	1 (0.002)					
10					1 (0.010)	
Total	566	28	227	27	100	28
EH	0.253	0.135	0.330	0.401	0.185	0.069
FRAXAC1						
1	1 (0.002)	1 (0.036)				
2	1 (0.002)					
3	167 (0.295)	16 (0.571)	63 (0.292)	2 (0.074)	30 (0.300)	5 (0.179)
4	395 (0.698)	11 (0.393)	153 (0.708)	25 (0.926)	70 (0.700)	23 (0.821)
5	1 (0.002)					
6	1 (0.002)					
Total	566	28	216	27	100	28
EH	0.427	0.518	0.413	0.137	0.420	0.293
FRAXAC2						
3	4 (0.007)					
4	113 (0.200)	9 (0.321)				
4+	2 (0.004)					
5	9 (0.016)					
5+	11 (0.019)	5 (0.179)				
6	91 (0.161)	1 (0.036)				
6+	35 (0.062)	3 (0.107)				
7+	295 (0.521)	10 (0.357)				
Total	566	28				
EH	0.651	0.724				

EH, expected heterozygosity; FXS, fragile X syndrome.

**Table 2:** Diversity of FRAXA and flanking STRs in unaffected Chinese population compared with other groups

Population	FRAXA		DXS548		FRAXAC1		FRAXAC2	
	No. of alleles	EH	No. of alleles	EH	No. of alleles	EH	No. of alleles	EH
Chinese^1^	33	0.678	6	0.253	6	0.427	8	0.651
Indonesians^2^	32	0.711						
Japanese^3^	31	0.795						
Caucasians^4^	42	0.838	10	0.485	6	0.469	13	0.687
African Americans^5^	34	0.808	14	0.685	6	0.603	18	0.88

EH, expected heterozygosity.

1 This study: for FRAXA, *n* = 1692; for DXS548, FRAXAC1, and FRAXAC2, *n* = 566.

2 Faradz et al. (2000), *n* = 1062.

3 Otsuka et al. (2010), *n* = 1161.

4 Crawford et al. (2000a), *n* = 721.

5 Crawford et al. (2000a), *n* = 637.

Our analysis identified 33 distinct haplotypes (DXS548-FRAXAC1-FRAXAC2) in the Chinese population, and the most frequent haplotypes were 7-4-7+ (0.440), 7-3-4 (0.173), and 7-4-6 (0.141).

### The CGG repeat lengths and patterns in unaffected Chinese population

**Figure 2:**
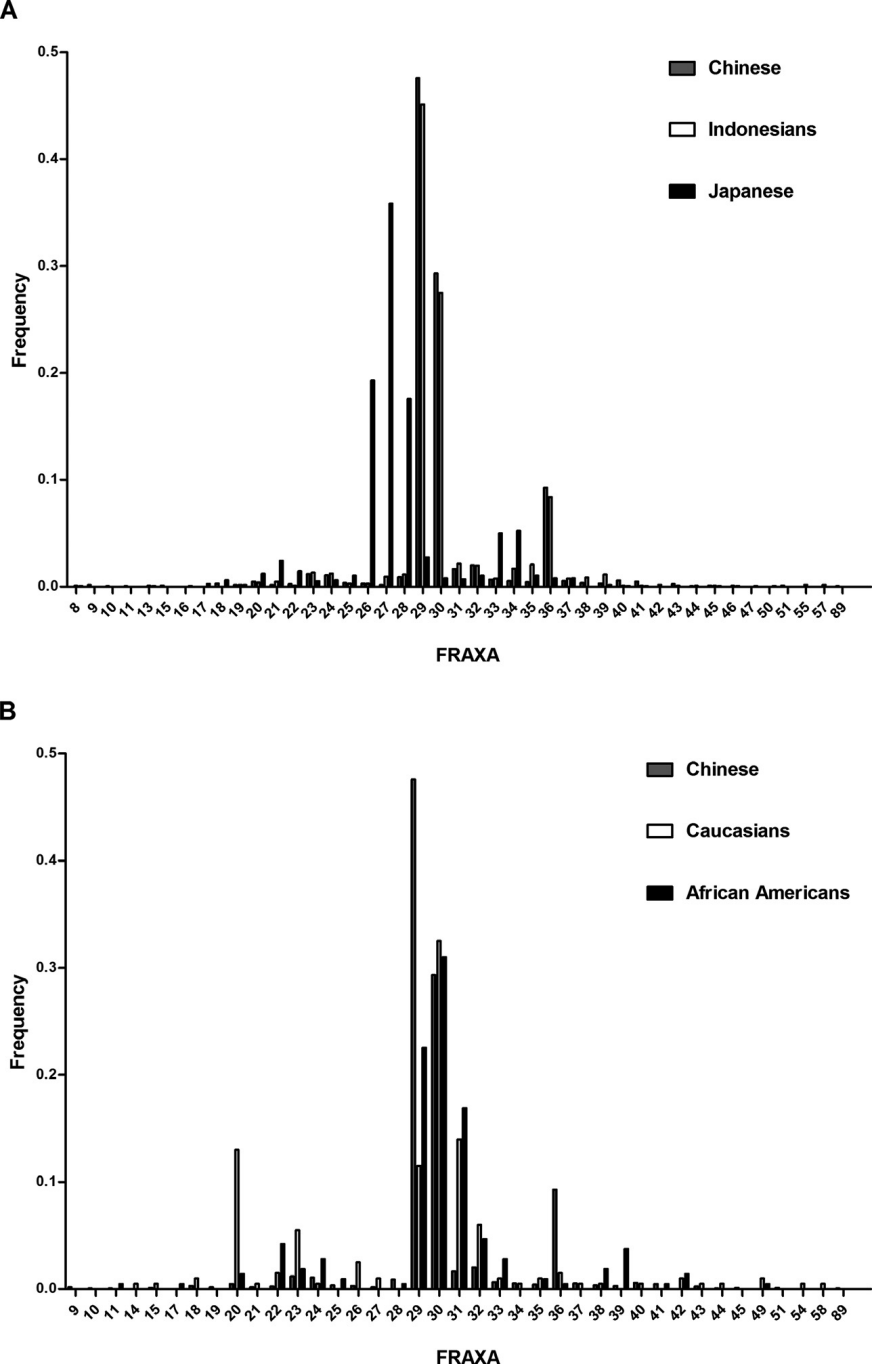
Frequency distribution of *FMR1* CGG repeat length among the unaffected populations. (A) Chinese (*n* = 1692; gray bars), Indonesians (*n* = 1062; white bars) (Faradz et al. 2000), and Japanese (*n* = 1161; black bars) (Otsuka et al. 2010). (B) Chinese (*n* = 1692; gray bars), Caucasians (*n* = 200; white bars) (Crawford et al. 2000b), and African Americans (*n* = 213; black bars) (Eichler et al. 1996). *FMR*, *fragile X mental retardation 1*.

Collectively, we analyzed 1113 unaffected Chinese individuals (534 males and 579 females) for CGG repeat lengths, and the CGG repeat patterns of 534 males were further determined by sequence analysis (32 males were excluded from the analysis of *FMR1* CGG repeat due to lack of DNA sample). We identified 33 different CGG repeat lengths on 1692 Chinese chromosomes (Table2). Chinese population has a similar number of CGG repeat lengths when compared with that of Indonesian and Japanese (Table2). The most frequent allele was (CGG)_29_ followed by (CGG)_30_ in the Chinese population (Fig.2A). Chinese population show both a smaller number of CGG repeat lengths and lower EH than Caucasians and African Americans (Table2). The distributions of the CGG repeat lengths were statistically different between the Chinese population and the other two populations (Chinese vs. Caucasians: *χ*^2^ = 230.2, *P *<* *0.0001, degrees of freedom [df] = 4; Chinese vs. African Americans: *χ*^2^ = 144.6, *P *<* *0.0001, df = 4). Allele (CGG)_30_ shows an almost identical or a slightly higher frequency compared with (CGG)_29_ in African Americans and Caucasians, respectively (Fig.2B). There was a second peak at (CGG)_36_ in the Chinese population, while a second peak between 20 and 22 repeats was observed in Caucasians and African Americans (Fig.2B). We only identified one premutation female carrier with 89 CGG repeats among the unaffected Chinese individuals of this study.

In the Chinese population, we found 62 different CGG repeat patterns on 32 distinct haplotypes (Table3). The CGG repeat pattern is shown as the number of pure CGGs followed by a (+) to indicate an AGG interruption (i.e., 9 + 9 + 9 represents (CGG)_9_AGG(CGG)_9_AGG(CGG)_9_). The most common patterns in the Chinese population were 9 + 9 + 9 (0.489), 10 + 9 + 9 (0.246), and 9 + 9 + 6 + 9 (0.074). The most frequent pattern in Caucasians is 10 + 9 + 9 (0.275), and the frequency of the 10 + 9 + 9 pattern (0.211) and the 9 + 9 + 9 pattern (0.211) are almost equal in African Americans (Eichler et al. 1996; Crawford et al. 2000b). The 9 + 9 + 6 + 9 pattern is absent in Caucasians and African Americans (Eichler et al. 1996; Crawford et al. 2000b). The majority of Chinese CGG repeat patterns had two AGG interruptions (0.788; Table3) in accordance with observations in Caucasians, African Americans, and Ghanaians, whereas the frequency of pure repeat alleles (0.015; Table3) in the Chinese population is the lowest compared with the other three populations (Eichler et al. 1996; Crawford et al. 2000b; Peprah et al. 2010). All of the intermediate alleles (45–54 repeats) in the Chinese population contain two or more AGG interruptions.

**Table 3:** Frequency of CGG repeat interspersion patterns among unaffected Chinese population (*n* = 534)

Interspersion	No.	Interspersion	No.	Interspersion	No.
Pattern	(Frequency)	Pattern	(Frequency)	Pattern	(Frequency)
9 + 9 + 9	262 (0.491)	8 + 9	1 (0.002)	10 + 6 + 9	1 (0.002)
10 + 9 + 9	130 (0.243)	9 + 6 + 6 + 9	1 (0.002)	10 + 7 + 9	1 (0.002)
9 + 9 + 6 + 9	39 (0.073)	9 + 6 + 9	1 (0.002)	10 + 9	1 (0.002)
9 + 19	13 (0.024)	9 + 8 + 9	1 (0.002)	10 + 9 + 6 + 9	1 (0.002)
19 + 9	7 (0.013)	9 + 9	1 (0.002)	10 + 9 + 10	1 (0.002)
9 + 13	4 (0.007)	9 + 9 + 6 + 6 + 9	1 (0.002)	10 + 9 + 13	1 (0.002)
10 + 19	4 (0.007)	9 + 9 + 6 + 7 + 9	1 (0.002)	10 + 10 + 9	1 (0.002)
9 + 9 + 16	3 (0.006)	9 + 9 + 7	1 (0.002)	10 + 13	1 (0.002)
9 + 10 + 9	3 (0.006)	9 + 9 + 7 + 9	1 (0.002)	10 + 17	1 (0.002)
9 + 14	3 (0.006)	9 + 9 + 9 + 9	1 (0.002)	10 + 21	1 (0.002)
9 + 20	3 (0.006)	9 + 9 + 10	1 (0.002)	11 + 9 + 9	1 (0.002)
9 + 21	3 (0.006)	9 + 9 + 11	1 (0.002)	12 + 9 + 9	1 (0.002)
9	3 (0.006)	9 + 9 + 12	1 (0.002)	13 + 9	1 (0.002)
9 + 12	2 (0.004)	9 + 9 + 15	1 (0.002)	14 + 9	1 (0.002)
9 + 16	2 (0.004)	9 + 9 + 19	1 (0.002)	19 + 6 + 9	1 (0.002)
9 + 23	2 (0.004)	9 + 9 + 31	1 (0.002)	20 + 9	1 (0.002)
9 + 24	2 (0.004)	9 + 11	1 (0.002)	21 + 9	1 (0.002)
10 + 9 + 11	2 (0.004)	9 + 11 + 9 + 9	1 (0.002)	11	1 (0.002)
10 + 20	2 (0.004)	9 + 15	1 (0.002)	15	1 (0.002)
12 + 6 + 9	2 (0.004)	9 + 16 + 9	1 (0.002)	19	1 (0.002)
18	2 (0.004)	9 + 25	1 (0.002)		

### Association between the CGG repeat patterns and flanking haplotypes

Our study shows that the FRAXAC1 alleles have a high association with the CGG repeat lengths. In the Chinese population, almost 73.6% of FRAXAC1 allele 4 were associated with (CGG)_29_, while 77.3% of FRAXAC1 allele 3 were associated with (CGG)_30_. Furthermore, we confirmed the presence of significant linkage disequilibrium between FRAXAC1 alleles and the CGG repeat patterns in the Chinese population. FRAXAC1 allele 4 strongly associates with the 9 + *n* pattern (*χ*^2^ = 367.0, *P* < 0.0001, df = 1), whereas FRAXAC1 allele 3 strongly associates with the 10 + *n* pattern (*χ*^2^ = 163.0, *P* < 0.0001, df = 1) in the Chinese population.

We determined the link between the CGG repeat patterns and flanking haplotypes in the present study. The patterns, 9 + 9 + 9 and 9 + 9 + 6 + 9, significantly associate with the 7-4-7 + haplotype, and the 10 + 9 + 9 pattern significantly associates with the 7-3-4 haplotype. The patterns 9 + 9 + 16 and 9 + 16 + 9 with the same length of the 9 + 9 + 6 + 9 pattern, were exclusively found on the 7-4-7 + haplotype.

### STR markers in Chinese fragile X population

Twenty-eight unrelated Chinese fragile X males were genotyped for DXS548, FRAXAC1, and FRAXAC2. For FRAXAC1 and FRAXAC2, our fragile X population have fewer alleles but higher EH when compared with our unaffected population (Table1). For DXS548, the fragile X population shows both fewer alleles and lower EH than the unaffected population (Table1). The most abundant alleles for DXS548 (allele 7) and FRAXAC2 (allele 7+) were identical between the fragile X and unaffected populations (Table1). With respect to FRAXAC1, the most frequent allele was allele 3 in the fragile X population, whereas the most frequent allele was allele 4 in the unaffected population. In previous fragile X samples from mainland China and Taiwan, the most common allele for DXS548 was allele 6 and allele 7, respectively, and both fragile X samples share the most common allele for FRAXAC1 (allele 3; Table1).

Our studies identified seven distinct haplotypes in the fragile X population. The most frequent haplotypes, 7-4-7+ and 7-3-4, were the same between the fragile X and unaffected populations. The 7-3-5+ haplotype accounted for 17.9% of all the fragile X chromosomes, which was significantly underrepresented in the unaffected population. The distribution of the 7-3-5+ haplotype differs statistically between the fragile X and unaffected populations (*χ*^2^ = 24.0, *P *<* *0.0001, df = 1).

## Discussion

Large-scale population studies have been helpful to identify the factors that may influence CGG repeat instability over the past decade (Eichler et al. 1996; Crawford et al. 2000a,b; Peprah et al. 2010). Han Chinese constitutes 92% of the population of mainland China and ∼19% of the entire global human population (Zhou et al. 2011). To date, no systematic analysis of the *FMR1* CGG repeat patterns and flanking haplotypes has been performed on such a large population, and limited studies have been conducted in Chinese fragile X population. To examine this, we performed a detailed analysis of the *FMR1* CGG repeat in a Han Chinese population, and a comprehensive survey of the flanking haplotypes in both Chinese unaffected and fragile X populations.

The most frequent alleles for DXS548 and FRAXAC1 were identical among our Chinese population, previous Chinese samples, and other Asians such as Indonesian, Japanese, and Thai (Richards et al. 1994; Zhong et al. 1999; Faradz et al. 2000; Limprasert et al. 2001; Tzeng et al. 2005). The distributions for both the CGG repeat lengths and patterns in the Chinese population share similarities to that in Singaporeans (Zhou et al. 2006). Among these two populations, the most frequent allele was (CGG)_29_ followed by (CGG)_30_, and the most commonly observed patterns were 9 + 9 + 9, 10 + 9 + 9, and 9 + 9 + 6 + 9. In this study, we identified several low abundance alleles for DXS548 and FRAXAC1 that have not been found in previous studies on mainland Chinese and Taiwanese (Zhong et al. 1999; Tzeng et al. 2005). Our analysis identified 33 distinct haplotypes (DXS548-FRAXAC1-FRAXAC2) in the Chinese population, and the most frequent haplotypes are 7-4-7+ (0.440), 7-3-4 (0.173), and 7-4-6 (0.141), which are similar to those of Singaporeans (Zhou et al. 2006). In total, 71 and 202 types of haplotypes have been determined in Caucasians and African Americans, respectively, and the most common haplotype is 7-3-4+ (Crawford et al. 2000a) that occurred on a single chromosome in the Chinese population.

Previous studies have demonstrated an insertion of (CGG)_6_ AGG in Asian populations such as Japanese, Singaporean, and Taiwanese (Chen et al. 1997; Hirst et al. 1997; Zhou et al. 2006; Chiu et al. 2008). Because the 9 + 9 + 9 pattern was the most common and putative ancestral structure in Asians, it has been proposed that the insertion of (CGG)_6_ AGG mainly occurs on the 9 + 9 + 9 pattern, producing the basic structure 9 + 9 + 6 + 9 that was prevalent in Asians (Chen et al. 1997; Hirst et al. 1997). The patterns 9 + 16 + 9, 9 + 9 + 16, and 9 + 15 + 9, share similar haplotype with the 9 + 9 + 6 + 9 pattern in Japanese population and these three patterns could have arisen from an A to C mutation or a deletion of the AGG interruption in the 9 + 9 + 6 + 9 pattern (Hirst et al. 1997). In the Chinese population of this study, the 9 + 9 + 6 + 9 pattern was prevalent, followed by the 9 + 9 + 16, 9 + 9 + 15, and 9 + 16 + 9 patterns, all of which share the same haplotype 7-4-7+. These patterns (9 + 9 + 16, 9 + 9 + 15, and 9 + 16 + 9) could have been generated from the 9 + 9 + 6 + 9 pattern in the same manner as in Japanese population (Hirst et al. 1997). The presence of (CGG)_6_ AGG in patterns like 10 + 9 + 6 + 9, 12 + 6 + 9, 19 + 6 + 9, 9 + 6 + 6 + 9, and 9 + 9 + 6 + 6 + 9 were also determined in this study, which were absent in the proposed evolutionary pathways in previous studies (Chen et al. 1997; Hirst et al. 1997; Chiu et al. 2008). The observations indicate that the insertion of (CGG)_6_ AGG may occur on a number of distinct CGG repeat patterns.

A previous study demonstrated founder effects on mainland Chinese, and the 6-4 haplotype (DXS548-FRAXAC1) accounted for almost 62.5% of all fragile X chromosomes and was significantly underrepresented in unaffected individuals (Zhong et al. 1999). We identified a novel haplotype 7-3-5+ (DXS548-FRAXAC1-FRAXAC2) that significantly associates with the full mutation.

We present here a comprehensive characterization of the *FMR1* CGG repeat in a large Chinese population. We also demonstrate a systematic haplotype analysis of Chinese unaffected and fragile X populations. The estimated premutation carrier frequency varies in terms of ethnic backgrounds, ranging from 1:251 in Spain, 1:246 in Finland, and 1:382 in the USA to 1:113 in Israel (Ryynanen et al. 1999; Toledano-Alhadef et al. 2001; Cronister et al. 2005; Fernandez-Carvajal et al. 2009). East Asians exhibit a relative lower prevalence of premutation carriers than the Caucasians. Only one premutation female carrier was found among 1113 unaffected Chinese individuals in this study. The premutation carrier frequency in Taiwan was 1:1674, and no premutation allele was found in 947 Japanese individuals (Tzeng et al. 2005; Otsuka et al. 2010). Additionally, we have summarized the FXS screening data among patients with intellectual disability and autism spectrum disorders in Chinese literatures since 1989. A total of 14,265 Chinese patients with intellectual disability in 53 studies were analyzed, 905 were identified as fragile X full mutation, representing a prevalence of 6.3%. 2.1% frequency of FXS in 894 Chinese patients with autism spectrum disorders were obtained through five studies, respectively (unpubl. data Li Yu (Yu L) & Ranhui Duan (Duan R)). The FXS prevalence among patients with intellectual disability and autism spectrum disorders presents a similar level as the Caucasians.

The development of genetic testing protocols and genetic counseling guidelines for FXS have just started in China. To date, 130 certified laboratories offering FXS test services (registered in the National Institutes of Health Genetic Testing Registry; available online at http://www.ncbi.nlm.nih.gov/gtr/) are located in 27 countries, and 68 certified laboratories are in the USA. There are 34 provinces in China, but only a few provincial-level hospitals provide genetic testing services for FXS (Li et al. 2013). Considering the premutation carrier frequency and imbalances in the regional development in China, at least one qualified laboratory with FXS genetic testing services in every province are needed to meet the projected demands. It is imperative to carry out large-scale *FMR1*mutation screening for Chinese Han and other ethnics, which may guide the establishment of genetic testing and counseling services, and eventually help the families affected with FXS in China.
